# Case Report: Beyond the ulcer: 51-month durability of left gastric artery embolization for obesity management in an antiplatelet-dependent, surgery-ineligible patient

**DOI:** 10.3389/fnut.2026.1762014

**Published:** 2026-05-13

**Authors:** Min Hu, Xiangrui Chen, Limei Zhou, Maolin Sun, Yunwei Han, Jingting Zhao

**Affiliations:** 1 Department of Dermatology, Zigong Third People's Hospital, Zigong, Sichuan, China; 2 Department of Oncology, Zigong Third People's Hospital, Zigong, Sichuan, China; 3 Interventional Radiology, Zigong Third People's Hospital, Zigong, Sichuan, China; 4 Publicity Department, Zigong Third People's Hospital, Zigong, Sichuan, China; 5 The Department of Oncology, Affiliated Traditional Chinese Medicine Hospital of Southwest Medical University, Luzhou, Sichuan, China; 6 Quality Management Office, Zigong Third People's Hospital, Zigong, Sichuan, China

**Keywords:** antiplatelet therapy, iatrogenic gastric ulcer, left gastric artery embolization, long-term outcomes, refractory obesity

## Abstract

We report a 51-month follow-up of transcatheter left gastric artery embolization (LGAE) in a 54-year-old male with central obesity (BMI 29.1 kg/m^2^, waist circumference 107 cm), post-stroke disability, osteoarthritis-induced immobility, compulsive binge eating, alcohol dependence, and clopidogrel-treated antiplatelet therapy—rendering conventional weight-loss interventions ineffective or contraindicated. Despite developing an 8-cm iatrogenic gastric ulcer at 30 days post-procedure, complete endoscopic healing was achieved with proton pump inhibitors and mucosal protectants. Sustained weight loss of 15.2% (from 84 kg to 71.2 kg) with 14.6 cm waist reduction correlated with resolution of knee pain (WOMAC score 68 → 12), sleep apnea, fatty liver (ALT 119 → 28 U/L), and dyslipidemia. This case demonstrates LGAE’s long-term efficacy in high-risk, surgery-ineligible obesity while highlighting the necessity of rigorous management for significant embolization-related complications, suggesting vascular remodeling capacity in gastric mucosa.

## 1 Introduction

Obesity, as a global pandemic, has affected over 2 billion people (1). Its management remains a significant challenge for populations with high comorbidity burdens (2, 3), particularly for patients with neurological sequelae and musculoskeletal comorbidities. This creates a therapeutic paradox: traditional lifestyle interventions often fail due to physical activity limitations and behavioral constraints, while bariatric surgery poses excessively high risks for patients requiring long-term antiplatelet therapy. This case presents a high-burden phenotype—a 54-year-old male (BMI 29.1, height 170 cm, weight 84 kg, waist circumference 107 cm)—with post-stroke claudication, osteoarthritis-induced mobility impairment, and poorly controlled metabolic syndrome (hypertension, dyslipidemia, obstructive sleep apnea). His compulsive binge eating (consuming four bowls of rice per meal plus nocturnal snacks) and alcohol dependence rendered dietary adjustments unfeasible. Current therapies lack effective solutions for such patients for whom “both caloric restriction and exercise interventions are nonviable,” highlighting an urgent need for minimally invasive alternatives that circumvent behavioral barriers while avoiding surgical risks.

Transcatheter left gastric artery embolization (LGAE) is a mechanistically rational approach that suppresses appetite by targeting ghrelin-secreting X/A-like cells in the gastric fundus (4–6). Preclinical studies confirm reduced circulating ghrelin post-embolization, and early human trials report 6.8% excess weight loss at 6 months (7). However, two critical knowledge gaps limit its clinical adoption: first, procedural safety concerns, particularly iatrogenic gastric ulcers from nonselective embolization (8); second, the absence of long-term efficacy data beyond 24 months in non-oncological populations. Existing literature predominantly originates from small cohorts with incomplete metabolic phenotyping, neglecting high-risk subgroups where surgical risks are amplified by polypharmacy (e.g., clopidogrel) or mobility impairments.

This case reports sustained weight control for 51 months despite early postoperative gastric ulceration. The patient developed a non-*Helicobacter pylori* giant ulcer at 30 days post-procedure but achieved complete endoscopic healing with proton pump inhibitor therapy. He maintained a 15.2% weight loss (from 84 kg to 71.2 kg), with resolution of obesity-related symptoms (knee pain, dyslipidemia, sleep apnea). Crucially, this outcome was attained in a patient contraindicated for metabolic surgery due to stroke history and antiplatelet dependence. By documenting the temporal associations among ulcer healing, weight trajectory, and symptom improvement over 51 months, this report provides one of the longest follow-up periods reported to date, supporting LGAE’s potential in complex obesity and offering practical insights for complication management in vulnerable populations.

## 2 Case description

**Figure 1:**
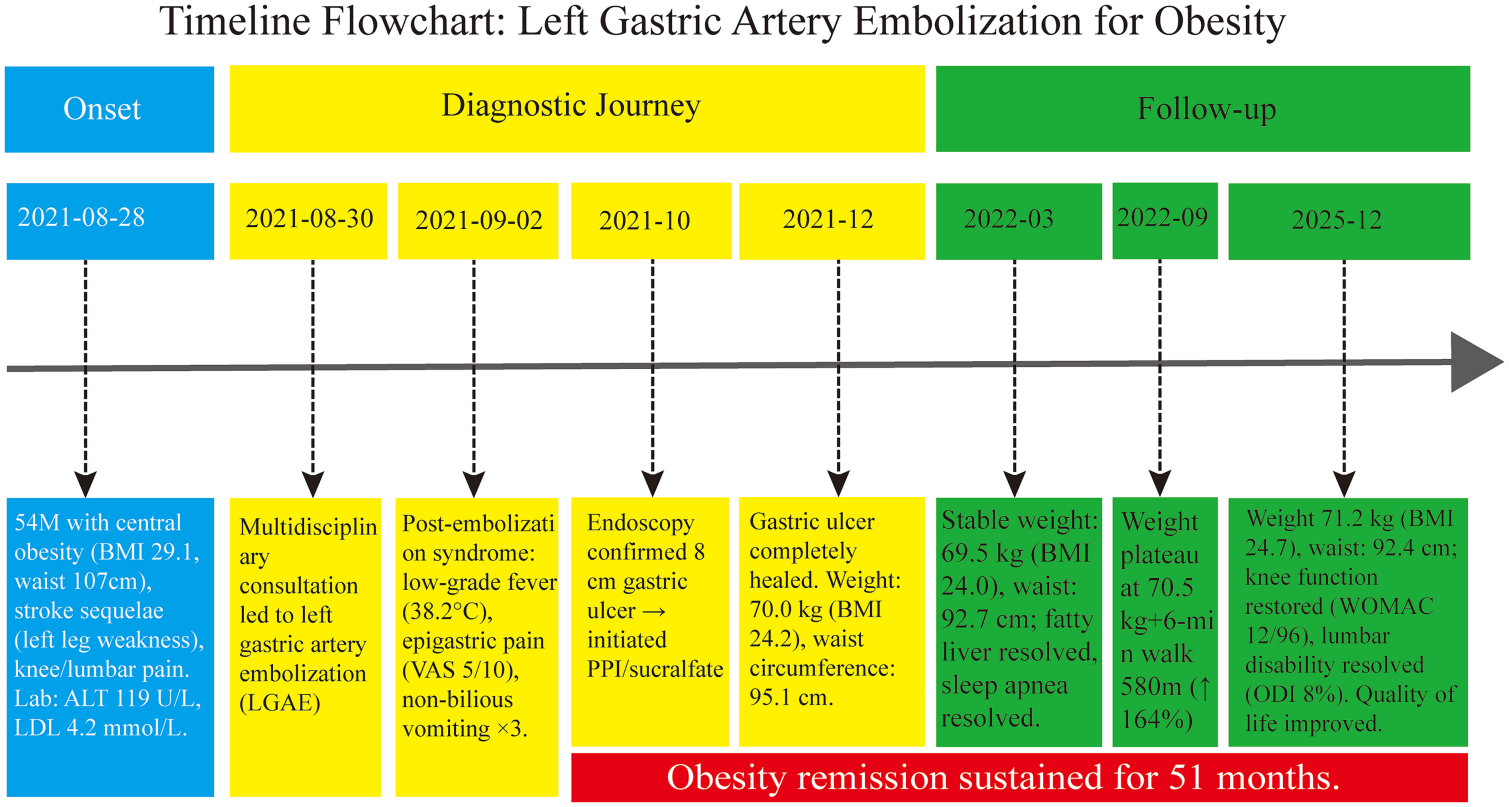
Timeline of left gastric artery embolization in the treatment of obesity. This figure illustrates the patient’s clinical course from admission to the last follow-up. The timeline is divided into three phases: Onset (blue), diagnosis and treatment (yellow), and follow-up (green). Key milestones include: (1) Multidisciplinary team consultation and decision to perform LGAE; (2) Post-embolization syndrome occurring on day 3 post-procedure; (3) Gastric ulcer confirmed by gastroscopy at 1 month post-procedure, with complete healing by 3 months; (4) Weight stabilization maintained from 6 to 24 months post-procedure; (5) Sustained obesity remission (BMI < 25 kg/m^2^) at 51 months post-procedure, accompanied by improved knee and lumbar function and quality of life.

All procedures involving human participants were strictly conducted in accordance with the ethical standards of the Ethics Committee of The Third People’s Hospital of Zigong City and the 1964 Helsinki Declaration and its later amendments. Written informed consent was obtained from the patient for publication of this case report and any accompanying images. The patient was followed from initial consultation on August 28, 2021, through December 2025, with key clinical milestones illustrated in Figure 1.

### 2.1 Clinical presentation and initial diagnosis

A 54-year-old Han Chinese male with central obesity presented to the Department of Oncology (including interventional oncology) at Zigong Third People’s Hospital on August 28, 2021, after learning that “obesity” could be treated via interventional methods. His medical history included ischemic stroke two years prior (resulting in left lower limb weakness and claudication), hypertension, dyslipidemia, and obesity. Clinical dietary interview documented compulsive binge eating: four bowls of rice per meal, high-calorie nocturnal snacking, and daily ethanol intake exceeding 60 g. Formal nutritional counseling was not pursued as the patient reported multiple prior failures with dietary interventions and specifically requested interventional therapy. Physical activity limitations due to lumbar disc herniation (L2/3, L4/5, L5/S1), bilateral knee osteoarthritis, and post-stroke gait instability and post-stroke lower limb motor incoordination rendered structured exercise programs intolerable due to exacerbation of joint pain. Consequently, traditional lifestyle-based weight management interventions involving structured exercise had failed repeatedly over three years due to poor adherence and physical constraints. Physical examination revealed stable vital signs (temperature 36.5 °C, blood pressure 158/92 mmHg, heart rate 85 bpm, respiratory rate 20/min, oxygen saturation 99%), a central obesity phenotype, height 170 cm, weight 84 kg (BMI 29.1 kg/m^2^), and waist circumference 107 cm—exceeding Chinese diagnostic criteria for obesity (BMI ≥ 28 kg/m^2^).

**Figure 2:**
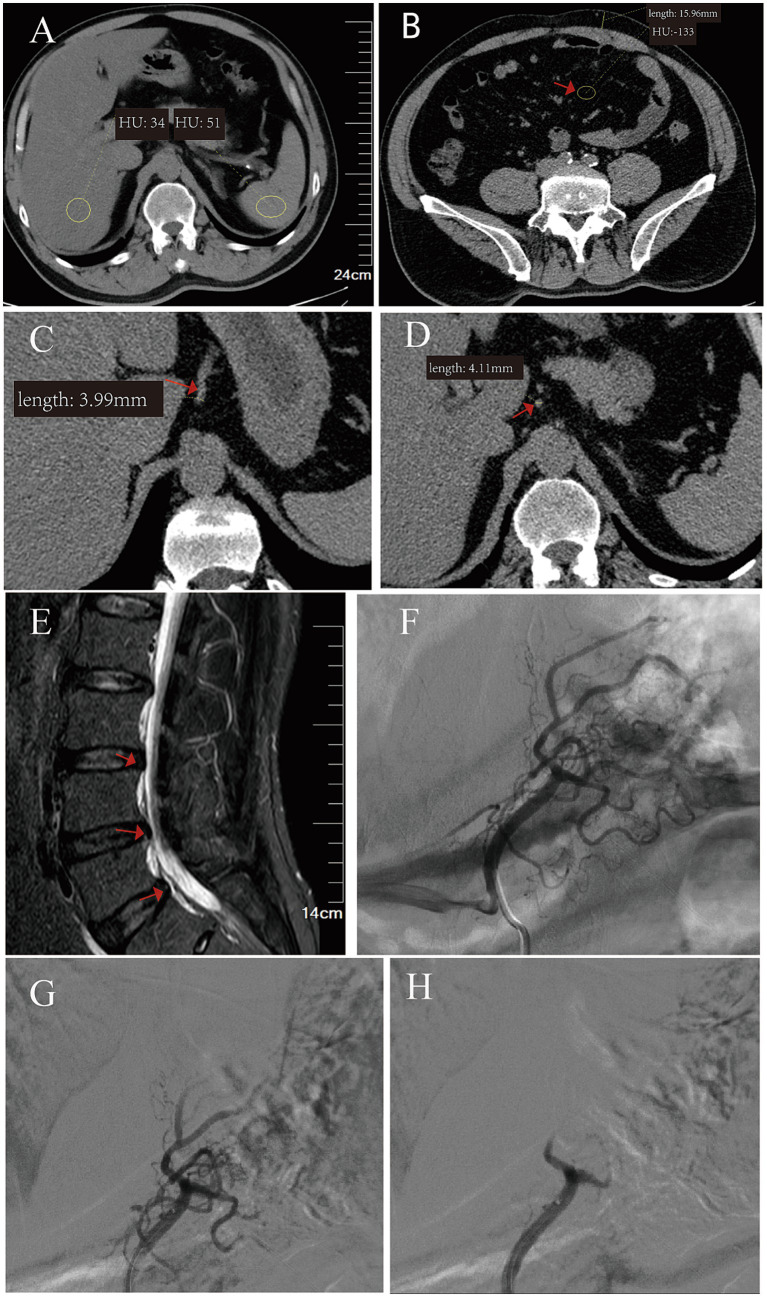
Pre- and post-procedural imaging findings of left gastric artery embolization for obesity. **(A)** Pre-procedure CT scan showing moderate hepatic steatosis. **(B)** Axial CT demonstrating increased abdominal wall fat thickness (15.96 mm) and mesenteric fat deposition (red arrow). **(C,D)** Coronal CT images measuring left gastric artery diameter: 3.99 mm **(C)** and 4.11 mm **(D)**, confirming suitability for superselective embolization. **(E)** Sagittal MRI revealing lumbar disc herniation at L4/5 and L5/S1 levels (red arrows). **(F)** Pre-embolization digital subtraction angiography: an enlarged left gastric artery with abundant fundal branches supplying the gastric body. **(G)** Post-embolization DSA demonstrating complete occlusion of the terminal branches. **(H)** Post-procedural DSA confirming the absence of residual flow in the main trunk.

Imaging findings included: Pulmonary: Emphysema with bilateral apical bullae and mild bronchiectasis; Abdominal: Moderate hepatic steatosis (Figure 2A), maximum abdominal fat thickness of 15.9 mm (Figure 2B), abundant mesenteric adipose tissue (Figure 2B), and left gastric artery dilation (Figures 2C,D); Musculoskeletal: Lumbar degenerative changes (L4/5 and L5/S1 disc bulging, Figure 2F), right knee degenerative changes with minimal effusion.

Laboratory tests (complete blood count, coagulation profile, hepatic/renal function, CRP) were within normal limits except for elevated LDL cholesterol (4.2 mmol/L), triglycerides (2.8 mmol/L), and transaminases (ALT 119 U/L, AST 67 U/L). Primary diagnoses included: obesity (central) with comorbidities of hyperlipidemia, fatty liver, and hypertension; additional diagnoses: ischemic stroke in recovery phase (with residual left lower limb motor dysfunction), lumbar disc herniation (L4/5, L5/S1) with lumbar degenerative disease, right knee degenerative disease, and pathological eating behavior (binge eating).

### 2.2 Multidisciplinary assessment and rationale for left gastric artery embolization

The patient’s uncontrolled metabolic comorbidities (central obesity with 107 cm waist circumference, fatty liver with elevated transaminases, hyperlipidemia) prompted multidisciplinary consultation (endocrinology, orthopedics, general surgery, neurology, interventional radiology). The decision was based on the following three key considerations.

Standard therapy contraindications: Long-term clopidogrel therapy (for secondary stroke prevention; HAS-BLED score 3) contraindicated bariatric surgery; severe lumbar disc herniation (L4/5, L5/S1) and right knee degeneration caused exercise intolerance. Prior attempts with over-the-counter weight-loss supplements (specific agents unrecalled), traditional Chinese medicine, and dietary restriction failed due to compulsive binge eating. Standard pharmacological interventions were discussed, but the patient expressed concerns regarding potential interactions with chronic alcohol use and antiplatelet therapy; additionally, the patient specifically requested interventional therapy after learning about minimally invasive “incision-free” weight-loss options available at our institution.

Obesity-driven comorbidity exacerbation: Orthopedic assessment confirmed knee pain (WOMAC score 68/96) and lumbar dysfunction (ODI 42%) were attributable to mechanical overload; fatty liver (ALT 119 U/L) and hypertension (158/92 mmHg) were directly linked to central obesity.

Anatomic suitability: Preoperative CT revealed a dominant left gastric artery (3.99 mm, Figure 2C) with rich vascular supply to the gastric fundus and no significant collateral circulation (later confirmed by angiography, Figure 2F).

The institutional metabolic disease committee designated LGAE as the preferred strategy for three reasons: (i) fundic embolization could selectively inhibit ghrelin secretion; (ii) the patient actively requested interventional therapy; (iii) short-term data supported this approach in high-risk populations (9).

### 2.3 Technical execution of left gastric artery embolization

The interventional procedure was performed on August 30, 2021, under local anesthesia with DSA guidance. Using a modified Seldinger technique, the right common femoral artery was accessed with a 5F micro-puncture set. A 5F Cobra catheter (Cook Medical, USA) was advanced over a 0.035-inch hydrophilic guidewire to the celiac trunk. Diagnostic angiography confirmed a dominant left gastric artery with extensive fundal supply (Figure 2F). Under roadmap guidance, a 2.7F Progreat microcatheter (Terumo, Japan) was coaxially advanced to the distal left gastric artery, avoiding branches to the lesser curvature.

Embolization was performed in two stages: (1) Distal embolization: Two vials (1 mL each) of 300–500 μm Embosphere microspheres (Merit Medical, USA) were injected until near-stasis of flow (angiography showed ~80% reduction in antegrade flow, Figure 2G). (2) Proximal reinforcement: Hand-cut gelatin sponge particles (1 mm in size) were injected to achieve complete flow stasis (Figure 2G).

Final angiography confirmed complete occlusion of the left gastric artery (Figure 2H) with patent splenic and common hepatic arteries. Total procedure time was 78 min, with 95 mL of contrast medium used. Hemostasis at the femoral puncture site was achieved by manual compression for 15 min.

### 2.4 Perioperative complications and management

Within 24 h, the patient developed classic post-embolization syndrome: low-grade fever (38.2 °C), epigastric pain (VAS 5/10), and three episodes of non-bilious vomiting. Laboratory tests showed transient elevations in CRP (48 mg/L) and amylase (186 U/L), with normal hepatic enzymes. Conservative management included intravenous hydration, ondansetron 8 mg IV every 8 h, and esomeprazole 40 mg IV twice daily. Symptoms gradually resolved by postoperative day 3, and the patient was discharged.

After discharge, the patient relocated to Guangzhou, China, for work. Appetite decreased by approximately two-thirds, but intermittent epigastric pain and mild vomiting (~2 episodes/day) persisted. At 30-day follow-up, he underwent gastroscopy at Foshan People’s Hospital (Guangzhou) due to persistent pain and vomiting, revealing an 8-cm giant ulcer with elevated margins on the lesser curvature of the gastric body. Histopathology confirmed chronic active gastritis without *H. pylori* infection or dysplasia. The ulcer was attributed to iatrogenic ischemia from vascular embolization. Treatment was intensified to: esomeprazole 40 mg orally twice daily, sucralfate 1 g four times daily; strict avoidance of NSAIDs, ethanol, and spicy foods. General dietary advice (small frequent meals, avoidance of gastric irritants) was provided during ulcer healing, but formal nutritional follow-up was not conducted due to the patient working in a different location. Repeat gastroscopy at 90 days showed complete epithelialization of the ulcer bed with no residual defect.

### 2.5 Long-term follow-up outcomes

**Figure 3:**
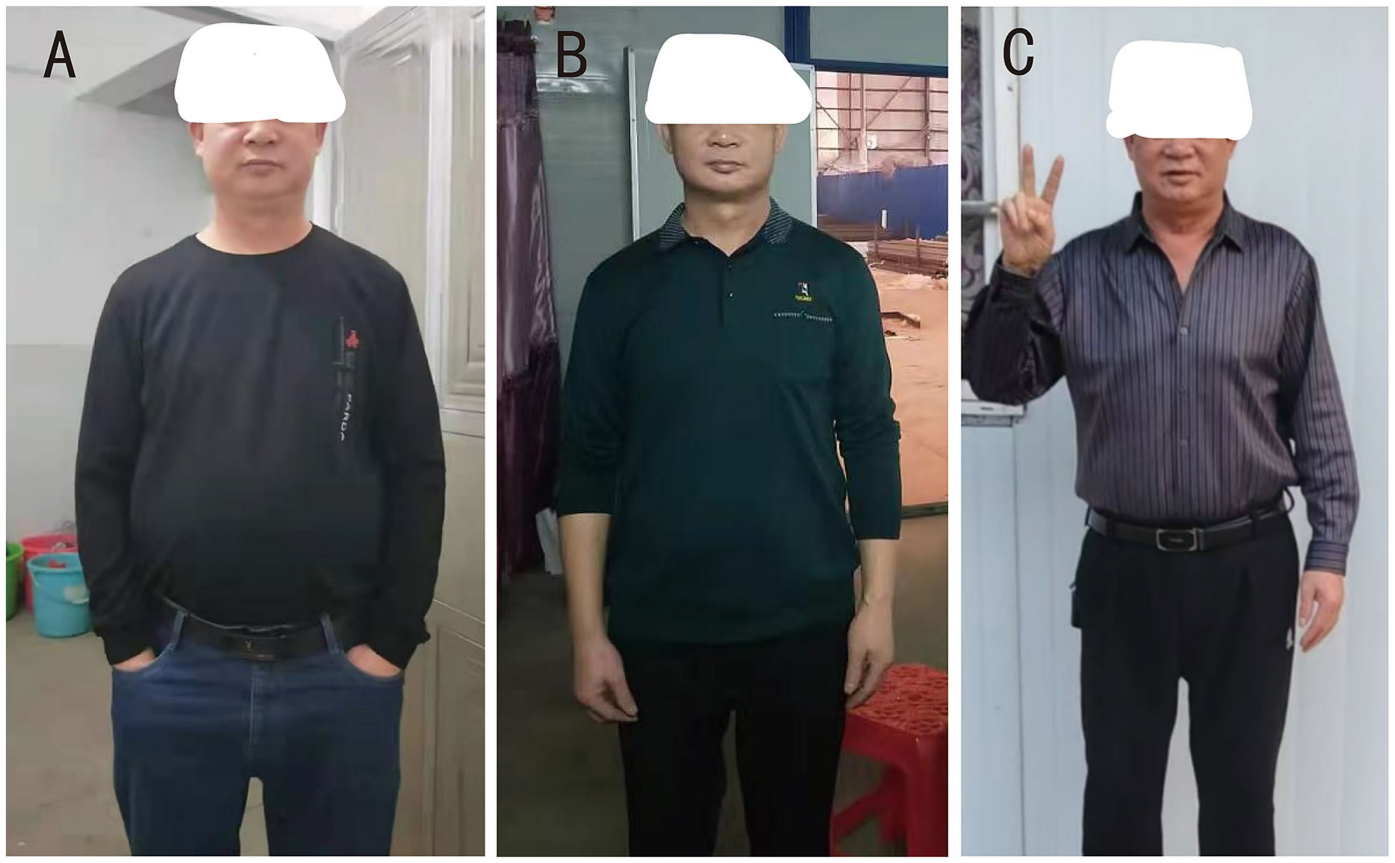
Pre- and post-procedure body appearance comparison of the patient. Clinical photographs demonstrating the patient’s body appearance changes during the 51 month follow-up period after left gastric artery embolization. **(A)** Pre-procedure baseline (August 2021): central obesity phenotype with prominent abdominal protrusion, weight 84 kg, BMI 29.1 kg/m^2^. **(B)** 3 months post-procedure (November 2021): significant reduction in abdominal girth following gastric ulcer healing, weight 70 kg, BMI 24.2 kg/m^2^. **(C)** 51 months post-procedure (December 2025): sustained weight maintenance with improved body contour, weight 71.2 kg, BMI 24.7 kg/m^2^. The patient reported improved mobility and quality of life, as demonstrated by the ability to wear previously ill-fitting clothing. Written informed consent was obtained from the patient for publication of these photographs.

The patient underwent clinical visits and metabolic assessments over 51 months. As detailed in Table 1, weight decreased progressively from 84.0 kg to 70.0 kg within the first 3 months, stabilizing at 71.2 kg by 51 months. Waist circumference followed a similar trajectory (107 → 92.4 cm). At the final follow-up (December 2025), the patient self-reported sustained resolution of obesity-related symptoms and provided pre- and post-procedure body appearance photographs for comparison (Figure 3), expressing high satisfaction with the outcome. The photographs demonstrate visible reduction in abdominal girth at 3 months post-procedure, with sustained weight maintenance at 51-month follow-up. Musculoskeletal: WOMAC knee score improved from 68 to 12/96; ODI lumbar dysfunction score improved from 42 to 8%. Cardiopulmonary: 6-min walk distance increased from 220 m to 580 m. Metabolic: Baseline hypertension (158/92 mmHg) was controlled to target ranges throughout follow-up with stable antihypertensive regimen; liver function normalized (ALT 28 U/L at 51 months) and lipids improved to normal range without lipid-lowering therapy. Sleep apnea symptoms resolved completely (patient-reported).

**Table 1:** Follow-up anthropometric, symptomatic, and metabolic parameters of the patient before and after left gastric artery embolization.

Time point	Weight (kg)	BMI (kg/m^2^)	Waist circumference (cm)	Key symptoms and signs	Metabolic parameters and key indicators
Pre-operation (2021-08-28)	84.0	29.1	107	Central obesity, mechanical pain in knees and waist (WOMAC 68/96, ODI 42%), limited mobility (6MWD 220 m).	Hypertension (158/92 mmHg), hyperlipidemia (LDL-C 4.2 mmol/L, TG 2.8 mmol/L), fatty liver (ALT 119 U/L, AST 67 U/L).
3 days post-operation	80.5	27.9	107	Acute procedure-related reactions: abdominal pain, vomiting.	Transient elevation of inflammatory markers (CRP 48 mg/L).
1 month post-operation	73.5	25.4	100	Intermittent complications: epigastric pain, vomiting (a large gastric ulcer confirmed by gastroscopy). Appetite reduced to one-third of the original level.	Blood pressure (128/67 mmHg, on medication); lipids (LDL-C 3.4 mmol/L, TG 2.1 mmol/L).
3 months post-operation	70.0	24.2	95.1	Gastric ulcer healed, resolution of abdominal pain and vomiting. Alleviation of knee pain.	Blood pressure (125/65 mmHg, on medication); lipids (LDL-C 3.1 mmol/L, TG 1.97 mmol/L).
6 months post-operation	69.5	24.0	92.7	Stable condition, no specific discomfort reported.	Blood pressure maintained within normal range on medication (122/62 mmHg).
12 months post-operation	70.5	24.4	91.4	Weight reached a plateau, condition remained stable.	Blood pressure maintained within normal range on medication (131/72 mmHg).
24 months post-operation	70.1	24.3	91.7	Long-term therapeutic effect was well maintained.	Blood pressure maintained within normal range on medication (121/55 mmHg).
51 months post-operation	71.2	24.7	92.4	Long-term weight maintenance (15.2% loss), knee pain resolved (WOMAC 12/96), lumbar function improved (ODI 8%), mobility improved (6MWD 580 m), sleep apnea resolved.	Blood pressure controlled on medication (128/71 mmHg), lipids improved to normal range, liver function normalized (ALT 28 U/L) without lipid-lowering therapy.

This table summarizes the longitudinal changes in key clinical parameters during a 51-month follow-up after left gastric artery embolization (LGAE). Data points include body weight, body mass index (BMI), waist circumference, patient-reported symptoms/signs, and critical metabolic indicators at pre-procedure baseline and multiple post-procedure time points. The most significant weight reduction and metabolic improvement occurred within the first 3 months post-LGAE, followed by long-term maintenance. A transient gastric ulcer complication occurred at 1 month and was completely resolved by 3 months with standard medical therapy. BMI, body mass index; LDL-C, low-density lipoprotein cholesterol; TG, triglycerides; CRP, C-reactive protein. Baseline blood pressure reflects untreated values; follow-up blood pressure reflects on-treatment measurements.

## 3 Discussion

This case provides early evidence of LGAE’s long-term feasibility in high-risk obese patients with post-stroke antiplatelet therapy. The patient was excluded from conventional weight-loss modalities due to clopidogrel dependence (HAS-BLED score 3), lumbar/knee degeneration, and compulsive binge eating (daily ethanol >60 g). LGAE avoided surgical bleeding risks and behavioral intervention barriers by selectively targeting ghrelin secretion in the gastric fundus. Although an 8-cm iatrogenic gastric ulcer developed postoperatively (attributed to over-embolization with 300–500 μm microspheres), representing a significant procedure-related adverse event, standardized PPI therapy combined with mucosal protectants achieved complete healing. This outcome underscores the critical importance of proactive complication monitoring rather than implying inherent procedural safety. This case suggests that precise superselective distal branch targeting combined with individualized embolization extent may help mitigate risks, offering a potential option for “dual failures” (diet/exercise nonresponders + surgical contraindications).

The mechanism of gastric ulceration requires reanalysis integrating anatomy and technical parameters. Although preoperative CT showed a dominant left gastric artery (3.99 mm), the vulnerability of terminal branches along the lesser gastric curvature was unrecognized. The two-stage embolization using 300–500 μm microspheres and gelatin sponge particles ensured weight-loss efficacy but induced localized ischemia by completely obstructing distal flow. Notably, ulcer healing hinged on three factors: (i) strict superselection of fundal branches (avoiding the lesser curvature); (ii) microsphere size >300 μm to prevent capillary penetration; (iii) 8 weeks of intensive acid suppression. The 51-month ulcer-free period confirms the gastric mucosa’s capacity for compensatory vascular remodeling. This finding may inform embolization strategy refinement: for high-risk patients, microspheres >500 μm or staged embolization should be used to preserve collateral circulation.

The 51-month follow-up data address a gap in LGAE’s long-term efficacy evidence, as most existing studies report follow-up periods of 6–24 months (5, 10). Although typical LGAE studies enroll patients with BMI > 40 kg/m^2^ (5, 10), this patient met Asian obesity criteria (BMI ≥ 28 kg/m^2^), supporting the applicability of LGAE in lower-BMI populations with high metabolic risk. A 15.2% weight loss coupled with a 14.6 cm waist circumference reduction (107 → 92.4 cm) yielded metabolic benefits extending beyond numerical changes: reversal of fatty liver (ALT 119 → 28 U/L), 82% WOMAC score improvement, and complete resolution of sleep apnea. These improvements are consistent with sustained ghrelin axis modulation proposed in prior studies. Animal studies indicate prolonged ghrelin reduction post-embolization (11), although direct ghrelin measurements were not performed in this case and the mechanism remains theoretical. Critically, the 164% increase in 6-min walk distance (220 → 580 m) markedly improved autonomy during post-stroke recovery. This outcome validates that >10% weight loss induces qualitative functional transformation in mechanically overloaded patients, offering an approach for multidisciplinary metabolic management.

Although this study confirms LGAE’s feasibility in an extremely complex obesity phenotype through ultra-long follow-up, its inherent limitations must be acknowledged. The single-case design cannot exclude confounding factors, such as the patient’s spontaneous post-procedure alcohol abstinence contributing to metabolic improvements, and unmeasured dynamic ghrelin changes weaken mechanistic inferences. Additionally, detailed nutritional data, such as serial dietary assessments or caloric intake quantification, were not systematically collected during follow-up. This was a retrospective case report where the primary outcome was weight change rather than dietary behavior modification, and the patient’s relocation for work limited consistent follow-up. However, this limitation also highlights the clinical reality that LGAE may be particularly valuable for patients who cannot engage with traditional nutritional interventions. Selective reporting of successful cases may also overestimate procedural safety; the 8-cm iatrogenic ulcer in this case precisely reveals the high risk of current embolization strategies in patients with vascular anatomical variants. More critically, while the 51-month follow-up is notable, it remains insufficient to assess the long-term stability of vascular remodeling and potential delayed complications, such as peristaltic dysfunction from gastric wall fibrosis. These limitations caution that LGAE’s clinical translation must rest on strict patient stratification, standardized complication prevention protocols, and multicenter registry studies quantifying the relationship between embolic particle size and safety. Future research would benefit from prospective designs integrating dynamic ghrelin axis monitoring and radiomics assessment to establish individualized treatment pathways for truly no-option patients while avoiding the pitfall of “trading complications for efficacy.”

## 4 Conclusion

Left gastric artery embolization offers a potential weight-loss solution for centrally obese patients with strict contraindications to surgery, though procedural risks such as gastric ulceration require careful monitoring with post-stroke antiplatelet therapy. Fifty-one-month follow-up confirms that despite early, curable gastric ulceration, a 15.2% weight loss and 14.6 cm waist circumference reduction yield significant metabolic improvements, providing a potential option for patients failing conventional therapies.

## Data Availability

The original contributions presented in the study are included in the article/supplementary material, further inquiries can be directed to the corresponding authors.
